# Effect of maternal postnatal balanced energy protein supplementation and infant azithromycin on infant growth outcomes: an open-label randomized controlled trial

**DOI:** 10.1016/j.ajcnut.2024.06.008

**Published:** 2024-06-24

**Authors:** Ameer Muhammad, Yasir Shafiq, Muhammad Imran Nisar, Benazir Baloch, Aneela Pasha, Nida Salman Yazdani, Arjumand Rizvi, Sajid Muhammad, Fyezah Jehan

**Affiliations:** 1 Vaccines and Other Initiatives to Advance Lives (VITAL) Pakistan Trust, Karachi, Pakistan; 2 Center of Excellence for Trauma and Emergencies and Community Health Sciences, The Aga Khan University, Karachi, Pakistan; 3 Global Advancement of Infants and Mothers (AIM), Department of Pediatric Newborn Medicine, Brigham and Women’s Hospital, Harvard Medical School, Boston, MA, United States; 4 Harvard T. H. Chan School of Public Health, Boston, MA, United States; 5 Center for Research and Training in Disaster Medicine, Humanitarian Aid and Global Health (CRIMEDIM), Università degli Studi del Piemonte Orientale “Amedeo Avogadro,” Vercelli, Italy; 6 Department of Pediatrics and Child Health Medical College, The Aga Khan University, Karachi, Pakistan; 7 Centre of Excellence in Maternal and Child Health, The Aga Khan University, Karachi, Pakistan

**Keywords:** balanced energy protein supplements, lactating mothers, azithromycin, exclusive breastfeeding, child growth, anthropometry

## Abstract

**Background:**

Maternal undernutrition is a direct risk factor for infant growth faltering.

**Objectives:**

We evaluated the effect of postnatal balanced energy protein (BEP) supplementation in lactating women and azithromycin (AZ) in infants on infant growth outcomes.

**Methods:**

A randomized controlled superiority trial of lactating mother–newborn dyads was conducted in Karachi, Pakistan. Mothers intending to breastfeed their newborns with mid-upper arm circumference of <23 cm and live infants between 0 and 6 d of life were randomly assigned to 1 of 3 arms in a 1:1:1 ratio. Lactating mothers in the control arm received standard-of-care counseling on exclusive breastfeeding, nutrition, infant immunization, and health promotion plus iron-folate supplementation until the infant was 6 mo old. In intervention arm 1, mothers additionally received two 75-g sachets of BEP per day. In intervention arm 2, along with the standard-of-care and BEP to the mother, the infant also received 1 dose of azithromycin (20 mg/kg) at the age of 42 d . The primary outcome was infant length velocity at 6 mo. The total sample size was 957 (319 in each arm).

**Results:**

From 1 August, 2018 to 19 May, 2020, 319 lactating mother–newborn dyads were randomly assigned in each arm, and the last follow-up was completed on 20 November, 2020. The mean difference in length velocity (cm/mo) between BEP alone and control was 0.01 (95% confidence interval [CI]: −0.03, 0.06), BEP plus AZ and control was 0.08 (95% CI: 0.03, 0.13), and between BEP + AZ and BEP alone was 0.06 (95% CI: 0.01, 0.11). There were 1.46% (14/957) infant deaths in the trial, and 17.9% (171/957) nonfatal events (injectable treatment and/or hospitalizations) were recorded.

**Conclusions:**

Postnatal maternal BEP supplementation and infant AZ administration could modestly improve infant growth outcomes at 6 mo, suggesting potential benefits in simultaneously addressing maternal and infant undernutrition.

This trial was registered at clinicaltrials.gov as NCT03564652.

## Introduction

Global estimates on malnutrition suggest that nearly 149.2 million (22%) of children under 5 experience stunted growth, preventing them from achieving their full developmental potential [1]. According to the WHO, a child with stunting has a length-for-age z-score (LAZ) below the median by >2 standard deviations [2]. These children have a greater risk of ill health and premature death [3,4]. Furthermore, children with stunting tend to earn less in adulthood and attain a lower level of education [5,6]. A child’s growth is typically impaired during infancy, and after 2 y the impairment is most likely permanent [7].

The sustainable development goals have specified a 40% reduction in the number of children under 5 who have stunting and a 30% reduction in low birth weight by 2025 [8]. Improving maternal nutrition during pregnancy and lactation is a key pathway toward this goal, directly impacting low birth weight and small for gestational age, and also subsequent growth faltering [9]. The WHO advocates for exclusive breastfeeding (EBF) during the initial 6 mo to ensure infants receive essential nutrients and bioactive components for their health, growth, and development [10,11]. The nutritional status of a lactating mother does not affect breast milk volume or protein levels, but her diet does affect the quality of fatty acids in it [12]. Certain micronutrients in breast milk, such as water-soluble vitamins and minerals, can be affected by the diet of a mother [12]. There has been minimal evidence that promoting EBF by itself is effective in enhancing infant linear growth in low- and middle-income countries (LMICs) [13]. Providing nutritional support to breastfeeding mothers might be a crucial step to enhancing their health and reducing childhood malnutrition [14].

In Pakistani mothers, short stature and low BMI (in kg/m^2^) are significantly associated with underweight infants [15]. Infants under 6 mo remain vulnerable with a heightened risk of infection, mortality, poor growth, and later poor neurodevelopment outcomes [16]. However, the literature presents inconclusive evidence regarding the impact of maternal nutritional supplementation, such as balanced energy protein (BEP) and lipid nutrient supplements (LNS), on infant anthropometry at 6 mo of age [[17], [18], [19]]. This research gap needs further investigation, especially on the effects of BEP supplementation in malnourished lactating mothers.

Apart from nutritional interventions, recent evidence also suggests that prophylactic administration of azithromycin (AZ) can have a positive impact on infant growth, especially in LMICs where infections are a major cause of morbidity and mortality [[20], [21], [22]]. With regard to this, WHO has issued guidelines for the restricted use of mass distribution of AZ in newborns to decrease infection-related morbidity and mortality [23]. However, it remains unknown whether combining a single prophylactic dose of AZ with maternal BEP would be more effective in reducing the risk of growth faltering among infants of malnourished mothers during the postnatal period. We conducted a multiarm randomized controlled trial in malnourished lactating mothers and their infants, to evaluate the effect of BEP with or without a single dose of AZ on infant growth compared with standard-of-care alone in promoting infant growth at 6 mo of age.

## Methods

### Trial design

This was a community-based, 3-arm, open-label, assessor-blinded randomized controlled, superiority trial conducted in 3 peri-urban communities of Karachi, Pakistan. The 2 interventions that were used in this trial, BEP and AZ, were procured locally. The manufacturers had no role in the design, conduct, or analysis of the study. The full trial protocol is published [24] and registered at clinicaltrials.gov (NCT03564652). The study was overseen by a technical committee and independent data and safety monitoring board. The protocol was approved by the Institutional Review Board (IRB) of VITAL Pakistan Trust, the Ethics Review Committee of the Aga Khan University, and the National Bioethics Committee of Pakistan. The trial was performed in accordance with the principles of the Declaration of Helsinki. We vouch for the completeness and accuracy of the reported data and the fidelity of the trial to the protocol.

### Participants

Lactating mothers were eligible for inclusion in the trial if they were malnourished based on mid-upper arm circumference (MUAC) of <23.0 cm and had delivered an infant within the past 7 d. Other inclusion criteria were the intention to remain in the catchment area and exclusively breast feed the infant for ≥6 mo. Exclusion criteria were the birth weight of infant <1500 g, known congenital anomaly, or serious illness [24]. Lactating mothers reporting allergies to any ingredient in the BEP sachet (peanut, lentils, chickpea, or dairy products) or those who were previously enrolled in the trial were excluded. Written informed consent was obtained.

### Treatment

Lactating mothers who were malnourished having a liveborn infant of 0–6 d were assigned in a 1:1:1 ratio to one of 3 arms and followed up until the infant was 6 mo old. Lactating mothers in the control arm received standard-of-care counseling on EBF, nutrition, infant immunization, and health promotion plus iron-folate supplementation until the infant was 6 mo of age, delivered through trained community healthcare workers via door-to-door visits. Mothers randomly assigned to intervention arm 1 (BEP alone arm) additionally received two 75-g sachets of BEP per day starting from the time of enrollment until the infant was 6 mo of age. In intervention arm 2, mothers received standard-of-care and BEP sachets, and the infant received 1 dose of prophylactic oral AZ (at 20 mg/km) at 42 d of life, with a window period of +7 d (BEP + AZ arm). The BEP supplement was a ready-to-use paste with premixed micronutrients; net weight per sachet was 75 g (energy 420 kilocalories; protein 10.5 g/sachet). It is manufactured locally by Ismail Industry, under the name of “Afzaaish” with protein derived from chickpeas, lentils, peanuts, and skimmed milk. Detailed product description is provided in Supplemental Table 1. This product is not commercially available and only accessible to implementing partners involved in nutrition interventions such as the World Food Program and various governmental and nongovernmental organizations. The premix used in the BEP product is different from the United Nations International Multiple Micronutrient Antenatal Preparation (UNIMMAP) by the addition of some minerals such as iodine, vitamin K, calcium, phosphorus, pantothenic acid, biotin, manganese, potassium, and magnesium, which are not included in UNIMMAP [25]. The dose of AZ was calculated using a digital application. The trained research staff reconstituted the suspension, administered the dose at home and the remaining suspension was archived for future reference. AZ administration on day 42 of birth was scheduled based on AZ safety profile among young infants as well as pragmatic feasibility as the 6-wk vaccination visit would also be on the same day. To assess the safety of the AZ prophylaxis dose, infants were monitored for 48 h postadministration. The research team recorded any side effects observed or reported, conducting follow-up visits 24 h apart. Adverse event data were submitted monthly to the Data and Safety Monitoring Board by the Principal Investigator, with fatal events reported to the Data and Safety Monitoring Board and IRB within 72 h of detection.

A computer-generated, stratified block randomization was conducted using StataCorp 2019 [26]. The randomization scheme was created independently by the trial statistician, with sizes of 3, 6, and 9. Self-adhesive, precoded sticky labels with unique identification numbers were applied to sealed opaque envelopes containing the coded randomization identification number and intervention name to ensure that the randomization process and allocation were concealed. The BEP supplement was provided for daily consumption by trial personnel at enrollment (anytime within 0–6 d of birth), daily for the first 15 d, every second day for 2 wk, every third day for the following 2 wk, and finally weekly until the child reached the age of 6 mo. However, research staff ensured that mothers in the intervention arms had a daily supply (dose of 2 sachets/d) of BEP intervention regardless of the frequency of visits. Empty sachets were collected, counted, and logged against the participant’s record to estimate adherence. At each visit, participants were also probed to determine if BEP was used by someone else and this was additionally documented for adherence. AZ doses to the infants were also administered by trial personnel. In case an infant missed an AZ dose at day 42 of birth, a window of 7 d was considered acceptable to administer the dose. Given the nature of the intervention, participants and the staff who were involved in dispensing and compliance of BEP and AZ were not blinded; however, outcome assessors and investigators were blinded.

### Data collection

Enrollment was done between 1 August, 2018, and 19 May, 2020, and follow-ups were completed by November 20, 2020. Participant data were recorded and stored in a logged dashboard that was closed 6 mo after the last participant enrollment. Each participant in the trial was followed up for 6 mo after enrollment with equal follow-up in all arms. Maternal and infant anthropometry measurements were taken at baseline and then every 4 wk until the infant reached the age of 6 mo. Maternal height was only assessed at baseline. Infant length, weight, head circumference, and MUAC were measured by trained personnel according to standardized procedures adapted from the INTERGROWTH-21st (International Fetal and Newborn Growth Consortium for the 21st Century) Project [27]. Each measurement was obtained independently by 2 personnel; for readings to be a valid agreement for the infant’s length was ±0.4 cm, the infant’s weight was ±20 g, the infant’s head circumference was ±0.4 cm, and the infant’s MUAC was ±0.4 cm. Further, valid agreement of maternal reading was ±0.5 cm for maternal MUAC, ±0.2 kg for maternal weight, and ±0.5 cm for maternal height. These allowable limits were derived from INTERGROWTH-21st and other best available evidence [27,28]. The data were then entered into a digital system that automatically calculated the average values. If the difference between measurements exceeded the allowable limits, the system would indicate the need for reassessment. Means of the final pair of values were used in analyses.

Adverse events were recorded at each visit, including illnesses that required hospitalization and/or death among participants or their infants. Study personnel followed the participants at home to assess adherence to the intervention, assurance of EBF, and assessment of danger signs. All assessments were recorded on pretested questionnaires, which had been translated into the local language. Data on maternal health, pregnancy history, birth outcomes, and household characteristics were collected at baseline. Postenrollment follow-up encounters included assessment of the infant’s health, feeding practices, infectious comorbidities, immunization as well as physical examination of infants.

### Outcomes

The primary outcome was mean infant length velocity (cm/mo) at 6 mo. The null hypothesis was non-superiority of BEP ± AZ to control; the alternative was superiority with the margin of clinical significance set at 0.12 cm/mo. The rationale for selecting length velocity as the primary outcome was to assess the impact on infant growth faltering.

Secondary outcomes of importance were mean infant weight velocity (g/kg/d), infant weight gain (g/month), LAZ, weight-for-age z-scores (WAZ), and weight-for-length z-scores (WLZ), all measured monthly until the infant reached 6-mo of age.

### Sample size

Limited data are available on the impact of BEP on infant length velocity during the first 6 mo of life. In a cluster-randomized controlled trial in rural Bangladesh, prenatal multiple micronutrient supplementation (MMN) was compared with iron and folic acid supplementation (IFA) supplementation. The difference in length velocity between MMN and IFA groups from birth to 18 months was small, measuring just 0.02 cm/mo [29]. Therefore, in the absence of clear evidence on the impact of BEP interventions, we hypothesized the effect size to be 0.12 cm/mo based on the assumption that a high dose of BEP used in the trial plus AZ in 1 of the arms would have an additional impact. The calculated sample size was 957 mother–infant dyads, with 319 in each group, assuming 80% power to detect the hypothesized difference using a one-tailed test with a 2.5% alpha level. Bonferroni correction was used to allow for multiple comparisons. The sample size accounted for a drop-out rate of 10% and infant mortality rate of 4% based on prior experience [[30], [31], [32], [33]].

### Statistical analysis

The primary analysis was by intention-to-treat performed for those who had anthropometry at 6-mo. All analyses were done using Stata, version 16, developed by StataCorp LLC [26]. Length velocity was calculated based on the formula: length velocity (cm/mo) = (length at 6-mo visit (cm) – length at enrollment (cm))/(age in days at 6 mo visit – age in days at baseline visit) × 30.4375. Similarly, weight was calculated as: weight gain (g/month) = (weight at 6-mo visit – weight at baseline)/(age in days at 6 mo – age in days at baseline visit) × 30.4375. Growth velocity was derived as per Patel exponential method: growth velocity (g/kg/d) = 1000 × ln (weight at 6-mo visit/weight at baseline)/weight at baseline/date of visit at 6 mo – date of enrollment [34]. The WHO 2009 Child Growth Standards were used to calculate other growth outcomes such as LAZ, WAZ, and WLZ [34]. Descriptive analysis of each arm was conducted, and percentages or continuous data with ± SD were reported. The baseline characteristics were assessed by each arm. Outcomes of mean length velocity, growth velocity, weight gain, z-scores, and change in z-score for 0–6 mo were compared using one-way analysis of variance (ANOVA). Tukey’s test was used for multiple comparisons. As determined a priori the analysis was adjusted for maternal age, MUAC (<21.0 cm and 21.0 cm or above), BMI (<18.5 kg/m^2^ and 18.5 kg/m^2^ or above), gravidity (<3 births and 3 or above), infant sex (male and female), birth weight (<2500 g and 2500 g or above), and length (in cm as continuous variable) using a general linear model. The decision to adjust for birth length in the analysis was driven by the inherent variability in the initial measurement timing, ranging from 0 to 6 days postbirth across the 3 study arms. This adjustment was recommended by the technical advisory committee, data safety and monitoring board, and senior statistician to control for baseline differences, ensuring that any observed effects on length velocity are due to the interventions rather than when the measurements were taken. Mixed model analysis was used to compare the change in outcome per month in both the intervention arms compared with the control arm. Applying repeated measures ANOVA, the analysis reported beta coefficients and 95% confidence intervals (CIs). To validate the ANOVA and Tukey post hoc tests used in our BEP trial, we confirmed normality for each group using Shapiro–Wilk tests, assessed homoscedasticity with Levene’s test to ensure equal variances, and verified independence through our study design. Additionally, we visually inspected data distributions with histograms and Q–Q plots to confirm that the assumption of normality was satisfied. The coefficients represented the rate of change in the outcome per month. For covariate adjustment, an unadjusted and stepwise adjusted model was run. Trajectories were plotted for mean length and weight by age for each arm, and monthly z-scores were also plotted.

## Results

### Participant characteristics

**FIGURE 1:**
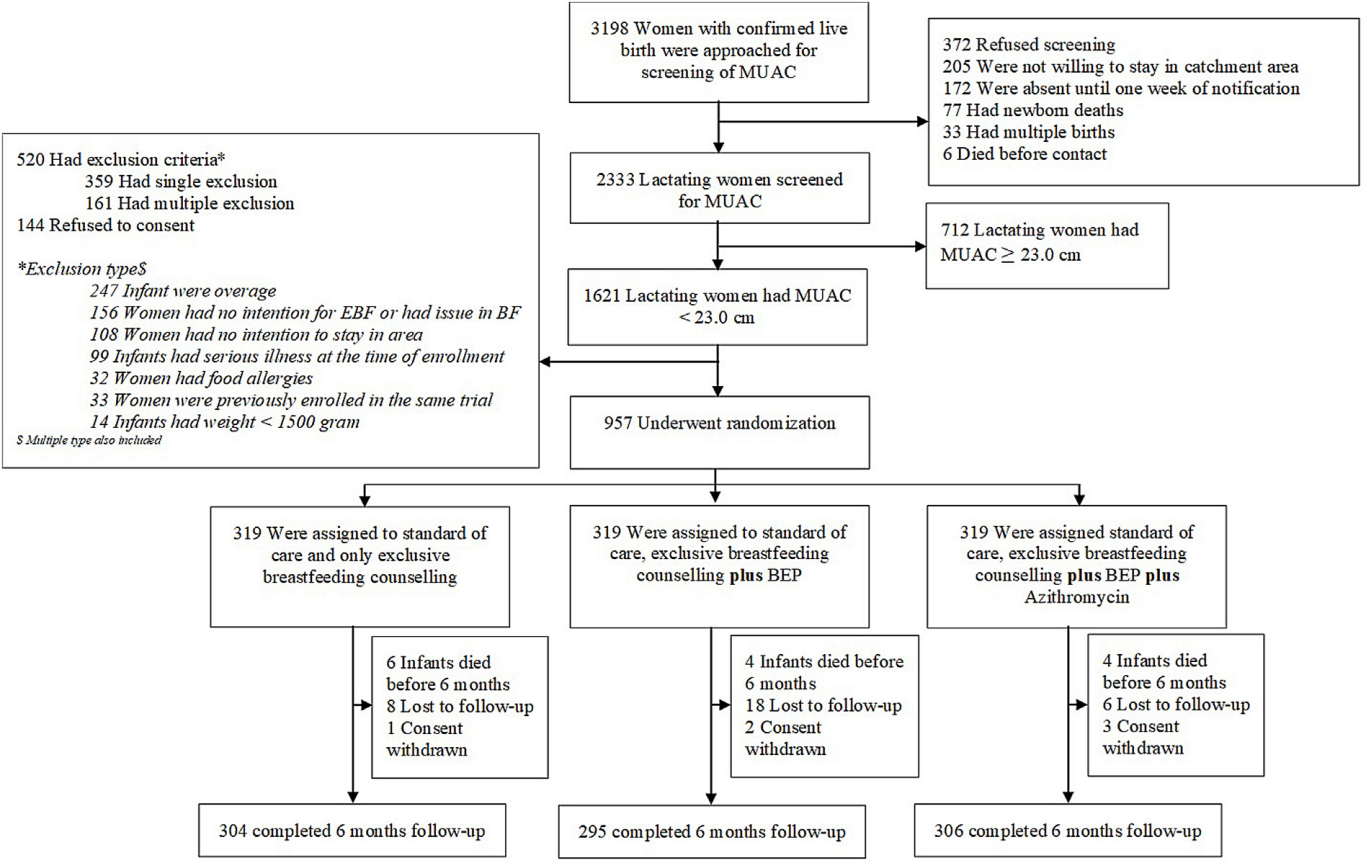
Flowchart of participants in the Mumta lactating woman trial. BEP, balanced energy protein; BF, breastfeeding; EBF, exclusive breastfeeding; MUAC, mid-upper arm circumference.

In the 2-y duration of the study, a total of 319 lactating mother–newborn dyads were randomly assigned per arm (Figure 1). Study arms were balanced about important variables at enrollment such as maternal age, education, MUAC, BMI, gravidity, parity, household characteristics, and infant anthropometric measures (Table 1). Early initiation of breastfeeding within 1 h of birth was low in the study, 31.9% (102/319) in the control arm, 36.9% (118/319) in BEP alone arm, and 32.9% (105/319) in BEP plus AZ arm. A higher proportion of lactating mothers had not discarded the colostrum, 95.0% (303/319) in control, 92.2% (294/319), and 91.5% (292/319) in the intervention arms, respectively. Overall, 26.1% (259/957) of mothers had a MUAC <21.0 cm and 19.8% (190/957) were underweight (BMI < 18.5 kg/m^2^) at enrollment. Low birth weight infants (<2500 g) were 30.8% (98/319), 27.3% (87/319), and 30.8% (98/319), respectively by arm.

**TABLE 1:** Baseline characteristics of mothers and infants enrolled in the Mumta LW trial.

Characteristics	Control arm (*n* = 319)	BEP alone arm (*n* = 319)	BEP plus azithromycin (*n* = 319)
Maternal characteristics^1^			
Age, mean (y)	24.4 ± 4.9	24.3 ± 4.9	24.1 ± 4.7
Anthropometry at baseline, mean			
Weight (kg)	45.8 ± 4.5	45.7 ± 4.5	45.5 ± 5.2
Height (cm)			
Body mass index (kg/m^2^)	19.7 ± 1.6	19.8 ± 1.6	19.6 ± 1.7
Mid-upper arm circumference (cm)	21.5 ± 1.1	21.5 ± 1.1	21.4 ± 1.2
Education, *n* (%)			
No education	194 (60.8)	198 (62.1)	203 (60.5)
Primary	54 (16.9)	50 (15.7)	46 (14.4)
Secondary	61 (19.1)	61 (19.1)	67 (21.0)
Intermediate or above	10 (3.1)	10 (3.1)	9 (4.1)
Obstetric history, median (IQR)			
Gravidity	3 (1–4)	3 (2–4)	3 (1–4)
Parity	2 (1–4)	2 (2–4)	2 (1–4)
Initiation of breastfeeding soon after birth, *n* (%)			
Colostrum given	303 (95.0)	294 (92.2)	292 (91.5)
Breastfeeding started within 1 h of birth	102 (31.9)	118 (36.9)	105 (32.9)
Prelacteal feeding given	224 (70.2)	224 (70.2)	220 (69.0)
Household characteristics, mean			
Household density^2^	4.9 ± 1.6	4.6 ± 2.0	4.5 ± 1.8
Material used for the floor of the house, *n* (%)			
Cement	292 (91.5)	292 (91.5)	296 (92.8)
Natural/mud	11 (3.4)	14 (4.4)	11 (3.4)
Wood	8 (2.5)	13 (4.1)	5 (1.6)
Tiles	8 (2.5)	–	7 (2.2)
Material used for the wall of the house, *n* (%)			
Cement	298 (93.4)	295 (92.5)	304 (95.3)
Natural/mud	5 (1.6)	4 (1.3)	4 (1.3)
Wood	10 (3.1)	19 (6.0)	7 (2.2)
Tiles	2 (0.6)	–	3 (0.9)
Temporary sheet	4 (1.3)	1 (0.3)	1 (0.3)
Source of drinking water, *n* (%)			
Access to improved drinking water	280 (87.7)	282 (88.4)	282 (88.4)
Water tanker	27 (8.5)	27 (8.5)	26 (8.2)
Community tap water	12 (3.8)	10 (3.1)	11 (3.4)
Maternal WHO food consumption score, mean	16.2± 2.8	16.4± 3.2	16.1± 2.8
Infant characteristics			
Demographic characteristics, *n* (%)			
Age at enrollment (d)	4.6 ± 1.6	4.4 ± 1.7	4.5 ± 1.6
Sex			
Male	160 (50.2)	149 (46.7)	153 (48.0)
Female	159 (49.8)	170 (53.3)	166 (52.0)
Anthropometry at birth, mean			
Length (cm)	48.0 ± 2.0	48.1 ± 2.0	47.9 ± 2.0
Weight (g)	2740 ± 405.5	2768 ± 398.6	2736 ± 432.7
Mid-upper arm circumference (cm)	9.3 ± 0.8	9.4 ± 0.8	9.4 ± 0.8
Head circumference (cm)	33.0 ± 1.2	33.0 ± 1.2	32. 9 ± 1.3
Length-for-age z-score	–1.0 ± 1.0	–0.9 ± 1.0	–0.9 ± 1.0
Weight-for-age z-score	–1.2 ± 1.0	–1.1 ± 1.0	–1.3 ± 1.0
Weight-for-length z-score	–1.4 ± 1.0	–1.4 ± 0.9	–1.4 ± 1.0
Malnutrition status, *n* (%)			
Birth weight			
≥2500 g	221 (69.2)	232 (72.7)	221 (69.2)
<2500 g	98 (30.8)	87 (27.3)	98 (30.8)
Wasting^3^	43 (13.4)	43 (13.4)	40 (12.5)
Stunting^4^	64 (20.0)	66 (20.6)	75 (23.5)
Underweight^5^	81 (25.3)	89 (27.8)	86 (26.9)
Immunization status, *n* (%)			
Fully vaccinated	183 (57.4)	187 (58.6)	183 (57.4)
Not vaccinated (zero doses received)	7 (2.2)	6 (1.9)	12 (3.8)

Abbreviations: BEP, balanced energy protein; LW, lactating woman.

Fully vaccinated means that the child received all 15 vaccines from birth to 9 months: BCG, OPV0, OPV1-3, ROTA 1-2, PENTA1-3, PCV1-3, IPV, and MCV1.

1 Plus–minus values are means ± SD.

2 Calculated as number of people per room in household.

3 Stunting was defined as a height-for-age z-score lower than −2 SD.

4 Wasting was defined as a weight-for-height z-score lower than −2 SD.

5 Underweight was defined as a weight-for-age z-score lower than −2 SD.

### Compliance with EBF and intervention

Overall, 72.4% (231/319) of infants in the control arm received EBF. In BEP alone and BEP plus AZ arms, EBF was 78.4% (250/319) and 74.6% (238/319), respectively. Compliance with BEP interventions among lactating mothers enrolled in BEP alone and BEP plus AZ was 93.5% and 91.9%, respectively (Supplemental Table 2).

### Outcomes

A priori adjustment for maternal age, MUAC, BMI, gravidity, infant sex, birth weight, and length showed the mean (±SD) length velocity (cm/mo) of infants was 2.77 ± 0.31 in controls, 2.78 ± 0.31 in BEP alone, and 2.85 ± 0.32 in BEP plus AZ arm. The mean difference in length velocity (cm/mo) between BEP alone and control was 0.01 (95% CI: –0.03, 0.06), BEP plus AZ and control was 0.08 (95% CI: 0.03, 0.13) and BEP plus AZ and BEP alone was 0.06 (95% CI: 0.01, 0.11) (Table 2). Among secondary outcomes, LAZ at 6 mo was –1.13 ± 0.80 in controls, –1.10 ± 0.81 in BEP alone, and –0.93 ± 0.81 in BEP plus AZ arm. The unadjusted analysis of the primary and secondary outcomes is presented in Supplemental Table 3.

**TABLE 2:** Outcomes at 6 months in the intention-to-treat analysis (adjusted).

	Control arm (*n* = 304)	BEP alone arm (*n* = 295)	BEP plus azithromycin arm (*n* = 306)	BEP alone vs. control		BEP plus azithromycin vs. control		BEP plus azithromycin vs. BEP alone	
				Mean difference (95% CI)	*P*	Mean difference (95% CI)	*P*	Mean difference (95% CI)	*P*
Primary outcomes									
Length velocity of infant (cm/mo)^1^	2.77 ± 0.31	2.78 ± 0.31	2.85 ± 0.32	0.01 (–0.03, 0.06)	0.558	0.08 (0.03, 0.13)	0.002	0.06 (0.01, 0.11)	0.014
Secondary outcomes									
Weight gain (g/mo)^2^	628.47 ± 134.34	641.30 ± 134.16	659.34 ± 134.26	12.83 (–8.7, 34.35)	0.243	30.87 (9.51, 52.22)	0.005	18.04 (–3.43, 39.51)	0.100
Growth velocity (g/kg/d)^3^	4.79 ± 0.79	4.85 ± 0.77	4.94 ± 0.77	0.06 (–0.07, 0.18)	0.357	0.16 (0.03, 0.28)	0.012	0.1 (–0.02, 0.22)	0.117
Change in length-for-age z-score per month	0.01 ± 0.14	0.02 ± 0.14	0.04 ± 0.14	0.01 (–0.02, 0.03)	0.644	0.03 (0.01, 0.06)	0.003	0.03 (0.01, 0.05)	0.012
Change in weight-for-age z-score per month	–0.03 ± 0.17	–0.01 ± 0.17	0.02 ± 0.17	0.02 (–0.01, 0.05)	0.205	0.04 (0.01, 0.07)	0.004	0.02 (–0.01, 0.05)	0.113
Change in weight-for-length z-score per month	–0.01 ± 0.20	0.01 ± 0.20	0.01 ± 0.20	0.02 (–0.01, 0.05)	0.256	0.02 (–0.01, 0.06)	0.142	0.01 (–0.03, 0.04)	0.747
Mid-upper arm circumference (MUAC) (cm) at 6 mo	12.84 ± 1.12	12.99 ± 1.12	13.07 ± 1.10	0.15 (–0.03, 0.33)	0.093	0.23 (0.06, 0.41)	0.010	0.08 (–0.1, 0.26)	0.372
Head circumference (cm) at 6 mo	40.96 ± 1.05	41.09 ± 1.05	41.06 ± 1.05	0.13 (–0.04, 0.3)	0.123	0.11 (–0.06, 0.27)	0.210	–0.03 (–0.19, 0.14)	0.767
Length-for-age z-score at 6 mo	–1.13 ± 0.80	–1.10 ± 0.81	–0.93 ± 0.81	0.03 (–0.1, 0.16)	0.656	0.2 (0.07, 0.32)	0.003	0.17 (0.04, 0.29)	0.011
Weight-for-age z-score at 6 mo	–1.53 ± 1.04	–1.42 ± 1.03	–1.28 ± 1.03	0.1 (–0.06, 0.27)	0.216	0.24 (0.08, 0.41)	0.004	0.14 (–0.03, 0.3)	0.103
Weight-for-length z-score at 6 mo	–1.02 ± 1.18	–0.91 ± 1.17	–0.87 ± 1.17	0.11 (–0.08, 0.3)	0.261	0.14 (–0.04, 0.33)	0.136	0.03 (-0.15, 0.22)	0.721

Abbreviations: BEP, balanced energy proteins; GLM, general linear model.

Data reported as mean ± SD or point difference (95% CI).

Outcomes are compared using one-way analysis of variance (ANOVA). Tukey's test was used for multiple comparisons.

The analysis is adjusted for the birth weight, birth length, maternal MUAC at enrollment, maternal age, gravidity, maternal BMI, sex of child, and respective baseline z-scores using general linear model (GLM).

*P* values are not adjusted for multiplicity.

1 Length velocity (cm/mo) = length at 6-mo visit − length at baseline/(date of visit of visit at 6 mo − date of enrollment) × 30.4375.

2 Weight gain (g/month) = weight at 6-mo visit − weight at baseline/(date of visit of visit at 6-mo − date of enrollment) × 30.4375.

3 Growth velocity (g/kg/d) defined as per Patel exponential method = 1000 × ln (weight at 6-mo visit/weight at baseline)]/weight at baseline)/date of visit at 6 mo − date of enrollment.

Among secondary outcomes, the mean difference in LAZ at 6 mo between BEP alone and controls was 0.00 (95% CI: –0.1, 0.16), BEP plus AZ and control was 0.20 (95% CI: 0.07, 0.32) and BEP plus AZ, and BEP alone was 0.17 (95% CI: 0.04, 0.29) (Table 2). Other secondary outcomes showed that the mean difference of weight gain (g/mo), growth velocity (g/kg/d), change in LAZ per month, change in WAZ per month, MUAC, LAZ, and WAZ at 6 mo were modest among BEP plus AZ arm compared with controls. The mean difference of change in LAZ per month and LAZ at 6 mo was also modest when comparing BEP plus AZ arm with BEP alone (Table 2). Further analysis showed that the rate of change of LAZ and WAZ had a positive slope among infants in the BEP plus AZ arm as compared with the control arm (Supplemental Table 4). Furthermore, infants in the control arm whose mothers had baseline MUAC < 21.0 cm had less mean length velocity than those whose mothers had baseline MUAC of 21.0 cm or greater. Detailed subgroup analyses are provided in Supplemental Tables 5 and 6. There were no differences in stunting, wasting, and underweight at 6 mo among the infants across arms (Supplemental Table 7). Growth trajectories over 6 mo showed that infants in BEP plus AZ arm fared relatively better overall, as compared with infants in control, and BEP only arm. (Supplemental Figure 1). There were 1.46% (14/957) infant deaths and 17.9% (171/957) nonfatal events (hospitalizations or injectable treatment) throughout the trial duration. About 1.9% (6/319) infant deaths were recorded in the control arm, 1.3% (4/319) in BEP alone arm, and 1.3% (4/319) in BEP plus AZ arm. Nonfatal events (hospitalizations or injectable treatment) were 20.7% (66/319) in the control arm, 16.6% (53/319) in BEP alone, and 16.3% (52/319) in BEP plus AZ arm. There was no maternal death reported and very few nonfatal serious adverse events were reported among lactating women (Table 3).

**TABLE 3:** Serious adverse events^1^.

Outcomes	Control arm (*n* = 319)	BEP alone arm (*n* = 319)	BEP plus azithromycin arm (*n* = 319)
Adverse event among infants, *n* (%)			
Infant deaths	6 (1.9)	4 (1.3)	4 (1.3)
Hospitalization and/or injectable therapy	66 (20.7)	53 (16.6)	52 (16.3)
Top 5 illnesses among infants			
Severe pneumonia	25 (7.8)	20 (6.3)	16 (5.0)
Presumed sepsis	22 (6.9)	14 (4.4)	16 (5.0)
Febrile illness	4 (1.3)	2 (0.6)	6 (1.9)
Diarrheal illnesses	11 (3.4)	10 (3.1)	7 (2.2)
Adverse event among women, *n* (%)			
Maternal deaths	0	0	0
Hospitalization and/or injectable therapy	4 (1.3)	2 (0.6)	1 (0.3)

Abbreviation: BEP, balanced energy proteins.

1 Denominator for each percentage per arm is 319.

## Discussion

In this randomized community-based trial of malnourished lactating mothers and their infants, we found that BEP supplementation to mothers in combination with a single dose of AZ to infants was effective in improving length velocity from baseline to 6 mo among infants as compared with control. However, BEP alone had no effect on infant growth. Importantly, in our trial, ≥20% of infants showed stunting and underweight at birth by WHO standards. Although secondary outcomes also showed improvement, the most important finding was the improvement in LAZ at 6 mo in both BEP plus AZ, as well as BEP alone arms, which may have notable implications for clinical decision-making.

EBF is recommended by WHO and UNICEF for the first 6 mo of life [10,11]. Maternal breast milk composition is heavily influenced by maternal nutrition status and diet [35]. Considering the central role of breastfeeding in infant growth, optimizing the nutritional status of mothers is essential [13,14]. There is a probable positive role of BEP in malnourished lactating women: first to provide adequate energy and nutrient consumption to meet maternal needs and second, to improve infant nutrition through optimizing breast milk composition. There is some prior evidence suggesting that high fat intake, >30% of the total caloric intake during pregnancy or postnatally, has a positive effect on the infant microbiome [36]. Although this was not the primary objective of our study, we found that the use of BEP was associated with a 0.3 cm increase in maternal MUAC at 6 mo (95% CI: 0.2, 0.4) (Supplemental Table 8 and Supplemental Figure 2). Mothers who received BEP gained more MUAC and experienced smaller decreases in BMI in the 6-mo postpartum period. Although BEP alone did not show any beneficial effects on infant growth, the use of 2 sachets of BEP substantially increased the fat stores of the breastfeeding mother. Different directions of MUAC and BMI trajectories postpartum have been reported and MUAC is lost during pregnancy followed by steady gains postpartum [37]. BMI, as expected, steadily declines postpartum given post-pregnancy weight loss [38].

Moreover, with recent evidence, there is an established role of antibiotics in the management of infection and malnutrition [[20], [21], [22]]. In a study of older Malawian children (6–59 mo) with severe acute malnourishment (SAM), antibiotic therapy along with ready-to-use therapeutic food improved nutrition recovery, mortality rate, and weight gain [39]. It is important to note that there are no optimal guidelines for the management of SAM in infants under 6 mo of age [40]. Although both amoxicillin and AZ have been used in the management of uncomplicated malnutrition, AZ is favored because it can be delivered as a single, oral dose due to its long half-life [41]. Further, in the large multicenter Macrolides Oraux pour Réduire les Décès avec un Oeil sur la Résistance (MORDOR) trial AZ reduced child mortality by 13.5% in children under 5 years of age in sub-Saharan Africa [21]. Although the precise mechanism behind this effect is unclear, it is likely due to an overall reduction in infectious burden [[42], [43], [44]]. With relevance to our trial, we postulate the role of AZ in alleviating infant dysbiosis and paving the way for improved uptake of nutrients from BEP fortified breast milk.

Data from LMICs on the effects of both BEP and AZ, separately and in combination, are sparse and inconclusive. Results from Burkina Faso (MISAME-III) published recently show that 6-mo growth outcomes of postnatal BEP supplementation did not differ significantly from the IFA-only group [18]. In contrast to our study, the BEP intervention in MISAME-III was a daily dose of 72-g sachet of BEP. A study in rural Malawi, which also investigated the effects of small quantity lipid-based nutrient supplement (20-g sachet) reported no effect on birth length at 18 mo [45]. It is possible that maternal and infant dysbiosis, left untreated, results in a modest effect of food-based interventions [45]. There have been other reports, for example, from Ghana [17] and Bangladesh [46] where BEP (or LNS) use resulted in a significant difference in growth at 18 mo of age. In both the studies, women in the intervention group received BEP supplements prenatally as well as postnatally and infants also received LNS supplementation from 6 to 18 mo of life. Adu-afarwuah et al. [17] reported mean LAZ at 6 mo as −1.30 ± 1.21, −1.19 ± 1.15, and −1.21 ± 1.05 among IFA, MMN, and LNS arms, respectively. However, the study did not report the mean differences in LAZ among different arms. Among Bangladeshi infants, Dewey et al. [46] reported differences in LAZ, WAZ, and WLZ in the LNS group at 18 mo. It is possible that the extended use of BEP resulted in sustained effects on fetal and infant linear growth.

Although the clinical margin of benefit may be low, our results indicate a positive signal in the face of an immensely challenging public health issue of malnutrition. The combined effect of maternal BEP supplementation during lactation along with AZ may also lead to a delayed positive effect on infant linear growth, weight gain, and mortality.

Noting the heterogeneity in BEP trials from the composition of supplements, duration of use and definition of comparator/control group, Gernand et al. [47] developed a meta-analyses framework for individual participant data across sites for a holistic and synchronized study on the effects of maternal BEP supplementation on pregnancy and postpartum outcomes for mother and child. The results of these meta-analyses will be better suited to inform decisions on BEP implementation in places where malnutrition is prevalent.

Notably, overall mortality rate between 0 and 6 mo within the trial cohort was 14.6/1000 live births (12.5/1000 live births in the intervention arms). This is significantly lower than Pakistan’s baseline neonatal mortality of 39.5/1000 live births and infant mortality of 62.5/1000 live births in 2016. However, the low number of deaths in the study cohort can be explained by rigorous follow-ups as well as the standard-of-care delivered by our research staff. It is also worth noting that mortality remained low in this cohort (overall infant mortality rate of 19.8/1000 live births) during extended follow-ups until 11 mo of age (15.6/1000 live births in the BEP alone and 21.9/1000 live births in BEP plus AZ arm).

The strengths of our trial include its randomized design and generalizability across LMIC settings. There was high compliance to the interventions in all arms, including EBF (Supplemental Table 3). Adherence to the nutrition intervention was assessed objectively through the collection of empty sachets and AZ administration was under direct supervision by the study team. The outcome assessment was done by staff that were trained, standardized, and blinded to the study arms. The trial arms were balanced with regard to the number of follow-up visits and the loss to follow-up rate was <10%. To date, this is the only study from an LMIC to evaluate the combined effects of postnatal BEP supplementation and AZ on growth outcomes. Our study supports the mechanistic pathway of interdependence between malnutrition, infection, and growth outcomes. Our trial also had important limitations, including the lack of a placebo for infants not in the AZ arm and the unblinded nature of the food supplement. During the first waves of COVID-19 in Pakistan (from 3 April 2020 to 31 August 2020), disruptions in health services and a lockdown created many challenges; however, with the support of the research team, provision of personal protective equipment, and careful scheduling of the teams, we were able to complete the randomization with a delay of only 3 wk. In addition, because most of our trained health workers were from the same community, routine follow-ups were not greatly compromised.

In conclusion, our results suggest that postnatal BEP supplementation to the mother, along with 1 dose of AZ to the infant is moderately beneficial in improving infant growth at 6 mo of age. Further research is needed to understand the association between breast milk composition and infant growth. It is important to assess how deficits in maternal nutrition translate to compromised infant growth. There is a need to understand the possible role of AZ in pregnancy, and if maternal gut microbiota modulation affects growth outcomes in infants. Long-term studies are warranted beyond 6 mo for the sustained effect of BEP and AZ, including patterns, mechanisms, and impact of bacterial resistance. Interventions to treat malnutrition in mothers together with dysbiosis in infants may have important implications for child growth.

When evaluating the interventions in our study, it is crucial to consider their cost-effectiveness alongside their statistical significance. Cost-effectiveness modeling studies of BEP supplementation during pregnancy [48] have demonstrated improvements in child morbidity and mortality under the age of 2, particularly when targeted at specific groups, such as undernourished women, rather than provided universally. This targeted approach can lead to better health outcomes for both mothers and children while optimizing resource allocation. In LMICs, the need for cost effective solutions is especially critical for interventions like BEP supplementation in lactating mothers. In many LMICs, these supplements are often used in emergency nutrition programs, and could be adapted for ongoing nutritional support to lactating mothers, potentially improving the quality of breast milk and infant growth trajectories [14]. However, a detailed cost-benefit analysis is essential but missing in many contexts. Such findings would be pivotal for policymakers aiming to optimize health outcomes against limited budgets, ensuring that interventions offered are scalable, sustainable benefits to public health in LMICs [49,50].

## Author contributions

The authors’ responsibilities were as follows – YS, FJ, MIN, AM: conceived and contributed to the design of the study and developed the study protocol and standard operating procedures; AM, NSY: implemented and supervised the study at the 4 sites; NSY: coordinated the activities and contributed to the manuscript; BB: provided coordination, medical advice, and support during the study; AM, AR, MS, AP: performed data analysis and interpretation, prepared tables, and figures; FJ, AM, YS, AP: drafted the manuscript; and all authors: read and approved the final manuscript.

## Conflict of interest

The authors report no conflicts of interest.

## Funding

The trial is funded by the Bill & Melinda Gates Foundation, grant number OPP1179727. The sponsor has no role or ultimate authority over any of the trial-related management, analysis, writing of the report, or the decision to submit the report for publication.

### Ethics approval and consent to participate

Ethics approval of the trial was obtained from the “Institution Review Board (IRB)” of VITAL Pakistan Trust (reference: 002-VPT-IRB-18 on 3 April, 2018), “Ethics Review Committee (ERC)” of Aga Khan University (reference: 5234-Ped-ERC-18 on 6 June, 2018), and “National Bioethics Committee (NBC)” of Pakistan (reference: 4-87/NBC-393/19/2170 on 29 May, 2019). All procedures performed in studies involving human participants were in accordance with the ethical standards of the institutional committee.

### Informed consent

Written informed consent was obtained from all participants and will be made available upon request.

### Consent for publication

The authors consent to the publication of this article. All authors have read and approved the final manuscript. This manuscript has not been published and is not under consideration for publication elsewhere.

### Patient public involvement

This research has been enriched by the active involvement of patients, their families, and members of the communities in which the study was conducted. We conducted regular community engagement activities to obtain their insights on our research objectives, outcomes, and methods. Locally hired and trained community health care workers from the study neighborhoods were involved in breastfeeding counseling and data collection. Their feedback and suggestions have played a pivotal role in shaping the research direction, enhancing the clarity of study materials, and ensuring that the study outcomes are meaningful to those it directly affects. We are grateful for their time, expertise, and dedication to advancing patient-centered research.

### Data availability

The data that support the findings of this study are available on request from the corresponding author.
