# Supplementary material for: Effect of maternal postnatal balanced energy protein supplementation and infant azithromycin on infant growth outcomes: an open-label randomized controlled trial
Source: Am J Clin Nutr. 2024 Jun 24;120(3):550–9. doi: 10.1016/j.ajcnut.2024.06.008 (PMC11393397; doi:10.1016/j.ajcnut.2024.06.008)
Supplement: Multimedia component 1 [file mmc1.docx]

| **Supplementary appendix. Ameer Muhammad et. al. Effect of maternal post-natal Balanced Energy Protein supplementation and infant Azithromycin on infant growth outcomes- An Open-label randomized controlled trial.**  **Supplementary Table 1 \| Composition of balanced energy protein (BEP) product (Afzaaish) with international standards ^ɸ^**   \| Ingredients \| Recommendation from report of an expert consultation held at the Bill & Melinda Gates Foundation (per day) \| \| Specification of BEP product (Afzaaish) used in the trial \| \| \| --- \| --- \| --- \| --- \| --- \| \| Per day \| \| 75-gram sachet \| Dose in 2 sachets \| \| Energy kcal \| 250 \| 500 \| 400 \| 800 \| \| Carbohydrate (g) \| No specific recommendations \| \| - \| - \| \| Protein (g) \| 14 \| 18 \| 10.5 \| 21 \| \| Fat (g) \| 10-60% of energy  Trans Fats: No more than 1%, as a standard safety requirement.  Minimum of 1.3 g of omega-3 fatty acids or 300 mg docosahexaenoic acid+ eicosatetraenoic acid (of which 200 mg DHA) to achieve a healthy omega-6 fatty acid: n ratio of the supplement of 5:1 \| \|  \|  \| \| Lipid total (g) \| 24 \| 48 \| \| Omega-6 fatty acids (g) \| 1.95 \| 3.9 \| \| Omega-6 fatty acids (g) \| 0.225 \| 0.45 \| \| Vitamin A (μg Retinol Equivalents) \| 550 \| 770 \| 42 \| 24 \| \| Vitamin D (μg) \| 10 \| 15 \| 112 \| 22.4 \| \| Vitamin E (mg) \| 16 \| 19 \| 12 \| 24 \| \| Vitamin K (μg) \| 72 \| 90 \| 20.2 \| 40.4 \| \| Thiamin (mg) \| 1.2 \| 1.4 \| 0.75 \| 1.5 \| \| Riboflavin (mg) \| 1.3 \| 1.6 \| 1.57 \| 3.14 \| \| Niacin (mg) \| 14 \| 18 \| 9.75 \| 19.5 \| \| Vitamin B6 (mg) \| 1.7 \| 2 \| 1.35 \| 2.7 \| \| Folate (μg) \| 400 \| 600 \| 27 \| 494 \| \| Vitamin B12 (μg) \| 2.4 \| 2.8 \| 2 \| 4 \| \| Vitamin C (mg) \| 100 \| 120 \| 45 \| 90 \| \| Calcium (mg) \| 500 \| 1000 \| 400 \| 800 \| \| Iron (mg) \| 22 \| 27 \| 7.5 \| 15 \| \| Zinc (mg) \| 15 \| 20 \| 8.2 \| 16.4 \| \| Iodine (μg) \| 209 \| 290 \| 75 \| 150 \| \| Biotin (μg) \| 28 \| 35 \| 5 \| 90 \| \| Pantothenic acid (mg) \| 5.6 \| 7 \| 3 \| 6 \| \| Choline (mg) \| 220 \| 550 \| - \| - \| \| Phosphorus (mg) \| 300 \| 700 \| 337 \| 674 \| \| Magnesium (mg) \| 145 \| 350 \| 112 \| 224 \| \| Manganese (mg) \| 2.1 \| 2.6 \| 0.9 \| 1.8 \| \| Copper (μg) \| 1 \| 1.3 \| 1000 \| 2000 \| \| Selenium (μg) \| 60 \| 70 \| 15 \| 30 \| \| Potassium (g) \| 2 \| 5.1 \| 0.675 \| 1.35 \| \| ^ɸ^ Bill & Melinda Gates Foundation. Framework and specifications for the nutritional composition of a food supplement for pregnant and lactating women (PLW) in undernourished and low‐income settings. Gates Open. Research. 2017.  kcal=kilocalories  gm=gram  mg=milligram  μg=microgram \| \| \| \| \|   **Supplementary Table 2 \| Cumulative compliance with exclusive breastfeeding and study interventions for 6 months period**   \|  \| Controls  N=319 \| BEP alone arm  N=319 \| BEP plus AZ  N=319 \| \| --- \| --- \| --- \| --- \| \| Exclusive breastfeeding compliance - n (%) $ \|  \|  \|  \| \| Exclusive breastfeeding \| 231 (72.4) \| 250 (78.4) \| 238 (74.6) \| \| Predominant/partial breastfeeding \| 88 (27.6) \| 69 (21.6) \| 81 (25.4) \| \| Compliance with BEP§ – Mean \|  \|  \|  \| \| Sachets received \| - \| 320.0 ±81.2 \| 325.6 ±74.2 \| \| Sachets consumed \| - \| 304.7 ±75.2 \| 308.8 ±64.9 \| \| Empty sachets returned \| - \| 289.2 ±78.3 \| 289.7 ±71.0 \| \| Sachet shared with family member \| - \| 0.4 ±2.2 \| 0.74 ±3.6 \| \| Percent compliance with BEP** \| - \| 93.5 \| 91.9 \| \| Compliance with Azithromycin^¶^ – n (%) \|  \|  \|  \| \| Administered within window period of 42-48 days \| - \| - \| 309 (96.9) \| \| Administered after window period \| - \| - \| 02 (0.6) \| \| Not administered-died before dose administration \| - \| - \| 02 (0.6) \| \| Not administered-refused for Azithromycin dose or lost-to-follow-up \| - \| - \| 06 (1.9) \| \| * Plus–minus values mean ± standard deviation.  ** Sachets consumed by the participants in each arm/sachet to be consumed by participants based on # of days in trial *100  § Only applicable to interventions arms which received BEP  ¶ Only applicable to intervention arm received Azithromycin  $ Breast feeding status is assess through 24 hours recall on schedule visit plus breast feeding status since past visit  ^1^Descriptive statistics for exclusive breastfeeding compliance and compliance with BEP and Azithromycin by treatment arm were calculated using the table1_mc command in Stata (version XX.X). The table includes the number of observations (N), percentages (%), and means with standard deviations (mean ± SD) where appropriate \| \| \| \|  \| **Supplementary Table 3 \| Infant growth outcomes at 6 months in the Intention-to-Treat analysis (Unadjusted)** \| \| \| \| \| \| \| \| --- \| --- \| --- \| --- \| --- \| --- \| --- \| \|  \| Controls N=304 \| BEP alone arm  N=295 \| BEP plus AZ arm  N=306 \| BEP alone Vs Control \| BEP plus Azithromycin Vs Control \| BEP plus Azithromycin Vs BEP alone \| \| Mean difference (95%CI) \| Mean difference (95%CI) \| Mean difference (95%CI) \| \| Primary outcomes \|  \|  \|  \|  \|  \|  \| \| Length velocity of infant (centimeters/month) ^a^ \| 2.77 ± 0.35 \| 2.78 ± 0.31 \| 2.85 ± 0.33 \| 0.01 (-0.06,0.07) \| 0.08 (0.01,0.14) \| 0.07 (0.01,0.13) \| \| Secondary outcomes \|  \|  \|  \|  \|  \|  \| \| Weight gain (grams/month) ^b^ \| 630.25 ± 143.22 \| 640.05 ± 141.47 \| 658.77 ± 142.47 \| 9.8 (-17.52,37.12) \| 28.52 (1.45,55.59) \| 18.72 (-8.55,46) \| \| Growth velocity (grams/kilograms) ^c^ \| 4.80 ± 0.90 \| 4.81 ± 0.89 \| 4.96 ± 0.89 \| 0.01 (-0.16,0.18) \| 0.16 (-0.01,0.33) \| 0.15 (-0.02,0.32) \| \| Change in Length-for-age z-score per month \| 0.01 ± 0.16 \| 0.01 ± 0.14 \| 0.05 ± 0.15 \| 0.00 (-0.03,0.03) \| 0.04 (0.01,0.06) \| 0.04 (0.01,0.06) \| \| Change in Weight-for-age z-score per month \| -0.02 ± 0.20 \| -0.01 ± 0.19 \| 0.02 ± 0.19 \| 0.01 (-0.03,0.05) \| 0.04 (0.01,0.08) \| 0.03 (0,0.07) \| \| Change in Weight-for-length z-score per month \| -0.01 ± 0.24 \| -0.00 ± 0.23 \| 0.01 ± 0.23 \| 0 (-0.04,0.05) \| 0.02 (-0.03,0.07) \| 0.01 (-0.03,0.06) \| \| Mid-upper-arm-circumference (centimeters) at 6 months \| 12.8 ± 1.2 \| 13.0 ± 1.2 \| 13.1 ± 1.1 \| 0.2 (-0.1,0.4) \| 0.2 (0,0.5) \| 0.1 (-0.2,0.3) \| \| Head circumference (centimeters) at 6 months \| 41.0 ± 1.4 \| 41.1 ± 1.4 \| 41.0 ± 1.5 \| 0.2 (-0.1,0.4) \| 0.1 (-0.2,0.3) \| -0.1 (-0.4,0.2) \| \| Length-for-age z-score at 6 months \| -1.15 ± 1.10 \| -1.06 ± 1.07 \| -0.95 ± 1.05 \| 0.09 (-0.12,0.29) \| 0.19 (-0.01,0.4) \| 0.11 (-0.1,0.31) \| \| Weight-for-age z-score at 6 months \| -1.54 ± 1.20 \| -1.39 ± 1.14 \| -1.29 ± 1.13 \| 0.15 (-0.07,0.37) \| 0.25 (0.03,0.47) \| 0.1 (-0.12,0.32) \| \| Weight-for-length z-score at 6 months \| -1.05 ± 1.17 \| -0.93 ± 1.14 \| -0.90 ± 1.17 \| 0.12 (-0.1,0.34) \| 0.14 (-0.08,0.37) \| 0.02 (-0.2,0.25) \| \| *Abbreviations, BEP: Balanced energy proteins, CI: confidence interval  Data reported as mean ± standard deviation (SD) or point difference (95% CI) \| \| \| \| \| \| \| \| Outcomes are compared using one-way analysis of variance (ANOVA). Tukey's test was used for the multiple comparisons. \| \| \| \| \| \| \| | | | | |
| --- | --- | --- | --- | --- | --- | --- | --- | --- | --- | --- | --- | --- | --- | --- | --- | --- | --- | --- | --- | --- | --- | --- | --- | --- | --- | --- | --- | --- | --- | --- | --- | --- | --- | --- | --- | --- | --- | --- | --- | --- | --- | --- | --- | --- | --- | --- | --- | --- | --- | --- | --- | --- | --- | --- | --- | --- | --- | --- | --- | --- | --- | --- | --- | --- | --- | --- | --- | --- | --- | --- | --- | --- | --- | --- | --- | --- | --- | --- | --- | --- | --- | --- | --- | --- | --- | --- | --- | --- | --- | --- | --- | --- | --- | --- | --- | --- | --- | --- | --- | --- | --- | --- | --- | --- | --- | --- | --- | --- | --- | --- | --- | --- | --- | --- | --- | --- | --- | --- | --- | --- | --- | --- | --- | --- | --- | --- | --- | --- | --- | --- | --- | --- | --- | --- | --- | --- | --- | --- | --- | --- | --- | --- | --- | --- | --- | --- | --- | --- | --- | --- | --- | --- | --- | --- | --- | --- | --- | --- | --- | --- | --- | --- | --- | --- | --- | --- | --- | --- | --- | --- | --- | --- | --- | --- | --- | --- | --- | --- | --- | --- | --- | --- | --- | --- | --- | --- | --- | --- | --- | --- | --- | --- | --- | --- | --- | --- | --- | --- | --- | --- | --- | --- | --- | --- | --- | --- | --- | --- | --- | --- | --- | --- | --- | --- | --- | --- | --- | --- | --- | --- | --- | --- | --- | --- | --- | --- | --- | --- | --- | --- | --- | --- | --- | --- | --- | --- | --- | --- | --- | --- | --- | --- | --- | --- | --- | --- | --- | --- | --- | --- | --- | --- | --- | --- | --- | --- | --- | --- | --- | --- | --- | --- | --- | --- | --- | --- | --- | --- | --- | --- | --- | --- | --- | --- | --- | --- | --- | --- | --- | --- | --- | --- | --- | --- | --- | --- | --- | --- | --- | --- | --- | --- | --- | --- | --- | --- | --- | --- | --- | --- | --- | --- | --- | --- | --- | --- | --- | --- | --- | --- | --- | --- | --- | --- | --- | --- | --- | --- | --- | --- | --- | --- | --- | --- | --- | --- | --- | --- | --- | --- | --- | --- | --- | --- | --- | --- | --- | --- | --- | --- | --- | --- | --- | --- | --- | --- | --- | --- | --- | --- | --- | --- | --- |
| **Supplementary Table 4 \| Mixed model analysis to compare change in growth outcome per month compared to the Controls** | | | |  |
| Outcome | Arm | Slope* (Arm*outcome) | 95%CI |  |
| Length | BEP alone | 0.0055 | -0.03, 0.05 |  |
|  | BEP plus AZ | 0.0664 | 0.03, 0.11 |  |
| Weight | BEP alone | 0.0086 | -0.00, 0.02 |  |
|  | BEP plus AZ | 0.0267 | 0.01, 0.04 |  |
| LAZ | BEP alone | -0.0004 | -0.01, 0.01 |  |
|  | BEP plus AZ | 0.0325 | 0.02, 0.05 |  |
| WAZ | BEP alone | 0.0052 | -0.01, 0.02 |  |
|  | BEP plus AZ | 0.0353 | 0.02, 0.05 |  |
| WHZ | BEP alone | 0.0046 | -0.02, 0.03 |  |
|  | BEP plus AZ | 0.0133 | -0.01, 0.04 |  |
| ^1^Mixed model analysis was conducted to compare the rate of change in growth outcomes per month between the intervention arms and the control arm. The analysis was performed using Stata 16, where beta coefficients and 95% confidence intervals (CI) were calculated. | | | | |
| *The coefficients represent rate of change in outcome per month. The sign of the coefficients shows direction of the effect | | | | |

**Supplementary Table 5 | Infant growth outcomes by sub-groups**

| Subgroups | | N | Controls  (A) | BEP alone arm  (B) | BEP plus AZ arm  (C) | C vs A | B vs C | B vs A | Arm A | Arm B | Arm C | C vs A | B vs C | B vs A |
| --- | --- | --- | --- | --- | --- | --- | --- | --- | --- | --- | --- | --- | --- | --- |
|  |  |  | Length velocity (centimetres/month) | | | | | | Weight gain (gram/month) | | | | | |
|  |  |  | Mean (±SD) | | | Mean difference (95%CI) | Mean difference (95%CI) | Mean difference (95%CI) | Mean (±SD) | | | Mean difference (95%CI) | Mean difference (95%CI) | Mean difference (95%CI) |
| Maternal MUAC | < 21.0 centimetres | 236 | 2.67 (0.32) | 2.73 (0.26) | 2.82 (0.31) | 0.15 (-0.00,0.29) | 0.09 (-0.06,0.22) | 0.06 (-0.08,0.21) | 592.96 (148.53) | 595.54 (121.68) | 657.05 (140.06) | 64.09 (-0.069,128.23) | 61.51 (-1.97,124.99) | 2.58 (-63.11,68.25) |
|  | ≥21.0 centimetres | 669 | 2.80 (0.39) | 2.79 (0.32) | 2.85 (0.34) | 0.05 (-0.03,0.14) | 0.06 (-0.02,0.15) | -0.01 (-0.10,0.07) | 642.24 (139.68) | 655.77 (144.83) | 659.43 (143.69) | 17.19 (-20.81,55.19) | 3.66 (-34.85,42.18) | 13.53 (-24.61,51.66) |
| Maternal BMI | <18.5 | 180 | 2.71 (0.35) | 2.78 (0.26) | 2.77 (0.31) | 0.06 (-0.10,0.23) | -0.01 (-0.18,0.15) | 0.07 (-0.10,0.25) | 594.85 (141.03) | 620.15 (133.89) | 659.14 (156.68) | 64.29 (-8.38,136.97) | 38.99 (-34.79,112.76) | 25.30 (-51.80,102.41) |
|  | ≥18.5 | 725 | 2.78 (0.34) | 2.77 (0.32) | 2.86 (0.33) | 0.08 (-0.00,0.16) | 0.09 (0.00,0.18) | -0.01 (-0.09,0.07) | 638.42 (142.75) | 644.50 (141.46) | 658.66 (138.41) | 20.24 (-16.67,57.16) | 14.16 (-22.98,51.30) | 6.08 (-30.67,42.85) |
| Birth weight | <2500 grams | 263 | 2.82 (0.40) | 2.81 (0.33) | 2.94 (0.35) | 0.12 (-0.02, 0.25) | 0.13 (-0.02, 0.26) | -0.01 (-0.15,0.13) | 627.37 (148.76) | 641.37 (144.00) | 650.28 (147.85) | 22.91 (-36.80,82.63) | 8.91 (-53.98,71.80) | 14.00 (-48.58,76.59) |
|  | ≥ 2500 grams | 642 | 2.74 (0.31) | 2.76 (0.30) | 2.81 (0.32) | 0.07 (-0.03,0.15) | 0.05 (-0.04,0.13) | 0.02 (-0.07,-0.10) | 631.54 (141.00) | 639.58 (140.88) | 662.42 (140.28) | 30.88 (-8.67,70.43) | 22.84 (-16.34,62.02) | 8.04 (-31.33,47.41) |
| Maternal age | <30 years | 742 | 2.79 (0.31) | 2.79 (0.29) | 2.85 (0.31) | 0.06 (-0.02,0.14) | 0.06 (-0.02,0.14) | 0.00 (-0.08,0.08) | 636.94 (135.76) | 646.87 (135.79) | 662.15 (137.77) | 25.21 (-11.09,61.51) | 15.28 (-20.94,51.50) | 9.93 (-27.12,46.97) |
|  | ≥ 30 years | 163 | 2.68 (0.43) | 2.70 (0.37) | 2.83 (0.40) | 0.15 (-0.04,0.32) | 0.13 (-0.06,0.32) | 0.02 (-0.16,0.18) | 605.63 (166.66) | 609.59 (162.33) | 638.61 (168.13) | 32.98 (-46.25,112.21) | 29.02 (-53.40,111.44) | 3.96 (-70.77,78.68) |
| Gravidity | <3 | 422 | 2.81 (0.33) | 2.80 (0.30) | 2.86 (0.31) | 0.05 (-0.05,0.16) | 0.06 (-0.04,0.17) | 0.01 (-0.10,0.12) | 642.86 (142.12) | 648.59 (141.43) | 669.06 (129.77) | 26.20 (-21.19,73.59) | 20.47 (-28.57,69.51) | 5.73 (-43.46,54.93) |
|  | ≥3 | 483 | 2.74 (0.36) | 2.75 0.32) | 2.83 (0.34) | 0.09 (-0.01,0.19) | 0.08 (-0.02,0.18) | 0.01 (-0.09,0.11) | 618.60 (143.68) | 633.50 (141.56) | 649.13 (153.19) | 30.53 (-15.17,76.25) | 15.63 (-29.45,60.73) | 14.90 (-30.19,59.99) |
| Gender | Male | 432 | 2.86 (0.30) | 2.85 (0.31) | 2.94 (0.34) | 0.08 (-0.02,0.19) | 0.09 (-0.01,0.20) | -0.01 (-0.12,0.09) | 673.20 (140.07) | 681.76 (138.69) | 708.20 (130.37) | 35.00 (-10.39,80.39) | 26.44 (-19.82,72.70) | 8.56 (-37.16,54.29) |
|  | Female | 473 | 2.68 (0.36) | 2.71 (0.29) | 2.76 (0.30) | 0.08 (-0.03,0.18) | 0.05 (-0.06,0.15) | 0.03 (-0.07,0.14) | 588.41 (133.94) | 602.88 (133.72) | 615.40 (138.80) | 26.99 (-16.65,70.63) | 12.52 (-30.97,56.03) | 14.47 (-29.65,58.58) |

*Arm A = Controls
 Arm B = BEP alone
 Arm C= BEP plus AZ

SD=standard deviation

CI=confidence interval

^1^Subgroup analysis was conducted to compare the mean differences in length velocity (cm/month) and weight gain (g/month) between the control arm and the two intervention arms merged (BEP alone and BEP plus Azithromycin). The analysis was stratified by maternal mid-upper arm circumference (MUAC), maternal body mass index (BMI), birth weight, maternal age, gravidity, and infant gender. The table reports mean values with standard deviations (Mean ± SD) for each subgroup, and mean differences with 95% confidence intervals (CI)

**Supplementary Table 6 | Infant growth outcomes by sub-groups ***

| Subgroups | | N | Controls  (A) | BEP alone arm  (B) | BEP plus Azithromycin arm  (C) | C vs A | B vs C | B vs A | Arm A | Arm B | Arm C | C vs A | B vs C | B vs A |
| --- | --- | --- | --- | --- | --- | --- | --- | --- | --- | --- | --- | --- | --- | --- |
|  |  |  | Length-for-age z-score at 6 months | | | | | | Weight-for-age z-score at 6 months | | | | | |
|  |  |  | Mean (±SD) | | | Mean difference (95%CI) | Mean difference (95%CI) | Mean difference (95%CI) | Mean (±SD) | | | Mean difference (95%CI) | Mean difference (95%CI) | Mean difference (95%CI) |
| Maternal MUAC | < 21.0 centimetres | 236 | -1.55 (1.10) | -1.36 (1.04) | -1.20 (1.07) | 0.35 (-0.12, 0.83) | 0.16 (-0.31, 0.64) | 0.19 (-0.30, 0.68) | -1.97 (1.34) | -1.82 (1.08) | -1.41 (1.07) | 0.56 (0.03, 1.08) | 0.41 (-0.09, 0.93) | 0.15 (-0.38, 0.67) |
|  | ≥21.0 centimetres | 669 | -1.01 (1.01) | -0.95 (1.06) | -0.85 (1.03) | 0.16 (-0.12, 0.44) | 0.1 (-0.19, 0.38) | 0.06 (-0.22, 0.34) | -1.40 (1.12) | -1.24 (1.12) | -1.25 (1.14) | 0.15 (-0.15, 0.46) | -0.01 (-0.32, 0.30) | 0.16 (-0.14, 0.47) |
| Maternal BMI | <18.5 | 180 | -1.45 (1.10) | -1.23 (1.05) | -1.31 (1.09) | 0.14 (-0.40, 0.68) | -0.08 (-0.63, 0.47) | 0.22 (-0.35, 0.79) | -2.01 (1.24) | -1.66 (1.06) | -1.40 (1.15) | 0.61 (0.01, 1.20) | 0.26 (-0.33, 0.85) | 0.35 (-0.27, 0.97) |
|  | ≥18.5 | 725 | -1.07 (1.08) | -1.02 (1.07) | -0.84 (1.02) | 0.23 (-0.04, 0.50) | 0.18 (-0.10, 0.45) | 0.05 (-0.22, 0.33) | -1.43(1.16) | -1.33 (1.14) | -1.26 (1.12) | 0.17 (-0.12, 0.47) | 0.07 (-0.22, 0.37) | 0.10 (-0.19, 0.39) |
| Birth weight | <2500 grams | 263 | -1.83 (1.07) | -1.88 (1.01) | -1.44 (1.12) | 0.39 (-0.02, 0.80) | 0.44 (0.00, 0.87) | -0.05 (-0.48, 0.38) | -2.14 (1.19) | -2.00 (1.20) | -1.86 (1.08) | 0.28 (-0.18, 0.74) | 0.14 (-0.34, 0.62) | 0.14 (-0.34, 0.62) |
|  | ≥ 2500 grams | 642 | -0.83 (0.96) | -0.76 (0.93) | -0.73 (0.95) | 0.10 (-0.17, 0.37) | 0.03 (-0.24, 0.30) | 0.07 (-0.20, 0.34) | -1.28 (1.10) | -1.18 (1.03) | -1.05 (1.06) | 0.23 (-0.08, 0.52) | 0.13 (-0.17, 0.42) | 0.10 (-0.20, 0.40) |
| Maternal age | <30 years | 742 | -1.03 (1.01) | -1.04 (1.05) | -0.93 (1.01) | 0.10 (-0.17, 0.36) | 0.11 (-0.16, 0.30) | -0.01 (-0.28, 0.27) | -1.45 (1.11) | -1.34 (1.09) | -1.29 (1.09) | 0.16 (-0.13, 0.45) | 0.05 (-0.24, 0.34) | 0.11 (-0.19, 0.40) |
|  | ≥ 30 years | 163 | -1.55 (1.30) | -1.13 (1.17) | -1.03 (1.29) | 0.52 (-0.06, 1.12) | 0.10 (-0.51, 0.72) | 0.42 (-0.14, 0.98) | -1.86 (1.44) | -1.60 (1.31) | -1.29 (1.31) | 0.57 (-0.83, 1.22) | 0.31 (-0.37, 0.98) | 0.26 (-0.34, 0.87) |
| Gravidity | <3 | 422 | -1.01 (0.96) | -1.10 (1.20) | -0.92 (1.00) | 0.09 (-0.27, 0.44) | 0.18 (-0.18, 0.55) | -0.09 (-0.46, 0.27) | -1.42 (1.14) | -1.40 (1.21) | -1.27 (1.02) | 0.15 (-0.23, 0.53) | 0.13 (-0.27, 0.52) | 0.02 (-0.37, 0.42) |
|  | ≥3 | 483 | -1.26 (1.20) | -1.02 (0.96) | -0.97 (1.09) | 0.29 (-0.04, 0.64) | 0.05 (-0.29, 0.38) | 0.24 (-0.09, 0.58) | -1.65 (1.24) | -1.39 (1.08) | -1.31 (1.22) | 0.34 (-0.03, 0.71) | 0.08 (-0.28, 0.44) | 0.26 (-0.10, 0.63) |
| Gender | Male | 432 | 1.23(1.09) | -1.19 (1.10) | -1.01 (1.06) | 0.22 (-0.14, 0.57) | 0.18 (-0.18, 0.54) | 0.04 (-0.33, 0.39) | -1.57 (1.22) | 1.43 (1.11) | -1.30 (1.14 ) | 0.27 (-0.11, 0.66) | 0.13 (-0.26, 0.52) | 0.14 (-0.24, 0.53) |
|  | Female | 473 | -1.06 (1.09) | -0.93 (1.03) | -0.89 (1.04) | 0.17 (-0.17, 0.51) | 0.04 (-0.30, 0.38) | 0.13 (-0.21, 0.47) | -1.51 (1.18) | -1.36 (1.16) | -1.28 (1.12) | 0.23 (-0.14, 0.59) | 0.08 (-0.29, 0.44) | 0.15 (-0.22, 0.52) |

*Arm A = Controls
 Arm B = BEP alone
 Arm C= BEP plus AZ

SD=standard deviation

CI=confidence interval

^1^Subgroup analysis was conducted to compare the mean differences in length-for-age z-score and weight-for-age z-score at 6 months between the control arm and the two intervention arms (BEP alone and BEP plus Azithromycin). The analysis was stratified by maternal mid-upper arm circumference (MUAC), maternal body mass index (BMI), birth weight, maternal age, gravidity, and infant gender. The table reports mean values with standard deviations (Mean ± SD) for each subgroup, and mean differences with 95% confidence intervals.

**Supplementary Table 7 | Infant nutrition indicators at 6 months**

| Outcomes | Control  N=304 | BEP alone arm  N=295 | BEP plus Azithromycin arm  N=306 | C vs A  (95% CI) | C vs B  (95% CI) | B vs A  (95% CI) |
| --- | --- | --- | --- | --- | --- | --- |
| Overall undernutrition (less than -2 SD) - n (%) | | | |  |  |  |
| Stunting | 63 (20.7) | 45 (15.3) | 47 (15.4) | -5.3 (-11.4, 0.8) | 0.1 (-5.7, 5.9) | -5.4 (-11.5, 0.7) |
| Underweight | 99 (32.6) | 72 (24.4) | 75 (24.5) | -8.1 (-15.3, -0.96) | 0.1(-6.8, 7.0) | -8.2 (-15.4, -1.0) |
| Wasting | 60 (19.7) | 48 (16.3) | 46 (15.0) | -4.7 (-10.7, 1.3) | -1.3 (-7.1, 4.5) | -3.4 (-9.5, 2.7) |
| Severe undernutrition (<-3SD) - n (%) | | | |  |  |  |
| Severe stunting | 13 (4.3) | 10 (3.4) | 8 (2.6) | -1.7 (-4.6, 1.9) | -0.8 (-3.5, 1.9) | -0.9 (-4.0, 2.2) |
| Severe underweight | 34 (11.2) | 25 (8.5) | 24 (7.8) | -3.4 (-8.1, 1.6) | -0.7 (-5.1, 3.7) | -2.7 (-8.1, 1.3) |
| Severe wasting | 18 (5.9) | 17 (5.8) | 16 (5.2) | -0.7 (-4.3, 2.9) | -0.6 (-4.3, 3.1) | -0.1 (-3.9, 3.7) |

*Arm A = Controls
 Arm B = BEP alone
 Arm C= BEP plus AZ

SD=standard deviation

CI=confidence interval

^1^Analysis was conducted to compare the prevalence of overall and severe undernutrition between the control arm, BEP alone arm, and BEP plus Azithromycin arm. The table reports the number and percentage of children with undernutrition outcomes, as well as the mean differences between the arms with 95% confidence intervals (CI)

**Supplementary Table 8 | Maternal BMI and MUAC over the period of 6 months**

| **Outcomes** | **Controls N=307** | **BEP** arms merged.**  **N=606** | **BEP** arms Vs controls** | |
| --- | --- | --- | --- | --- |
|  |  |  | **Mean difference (95% CI)** | **P-values** |
| Maternal outcomes |  |  |  |  |
| Change in BMI over 6-month (kilogramm^2^ per month) ^a^ | -0.20 ± 0.24 | -0.14 ± 0.26 | 0.05 (0.02,0.09) | 0.003 |
| Change in BMI at 6-month kilogramm^2^ ^b^ | -1.16 ± 1.14 | -0.86 ± 1.51 | 0.30 (0.10, 0.51) | 0.003 |
| Change in MUAC over 6-month (centimetres per month) ^c^ | 0.16 ± 0.25 | 0.22 ± 0.26 | 0.06 (0.02, 0.10) | <0.000 |
| Change in MUAC at 6-month centimetres ^d^ | 0.94 ± 1.44 | 1.32 ± 1.55 | 0.37 (0.16, 0.58) | <0.000 |
| *Abbreviations, BEP: Balanced energy proteins, CI: confidence interval, SD: Standard deviation | | |  |  |
| Data reported as mean ± SD or point difference (95% CI) |  |  |  |  |
| a Change in BMI Kilogramm2 per month) = (BMI at 6-month visit – BMI at baseline) / ((Date of visit of visit at 6-month – Date of enrolment) *30.4375) | | | |  |
| b Change in BMI at 6 months (Kilogramm2) = BMI at 6-month visit – BMI at baseline | | | |  |
| c Change in MUAC centimetres per month) = (MUAC at 6-month visit – MUAC at baseline)/ ((Date of visit of visit at 6-month – Date of enrolment) *30.4375) | | | |  |
| d Change in at 6 months (centimetres) = MUAC at 6-month visit – MUAC at baseline | | | |  |
| Outcomes are compared using one way analysis of variance (ANOVA).  ** BEP = Combined both intervention arms  ^1^Maternal outcomes were compared between the control arm and the combined BEP arms (BEP alone and BEP plus Azithromycin) over a 6-month period using one-way analysis of variance (ANOVA). The table reports the mean changes with standard deviations (Mean ± SD), mean differences with 95% confidence intervals (CI), | | |  |  |

**Supplementary Figure 1 | Comparison of LAZ, WAZ and WLZ trajectories over six months**


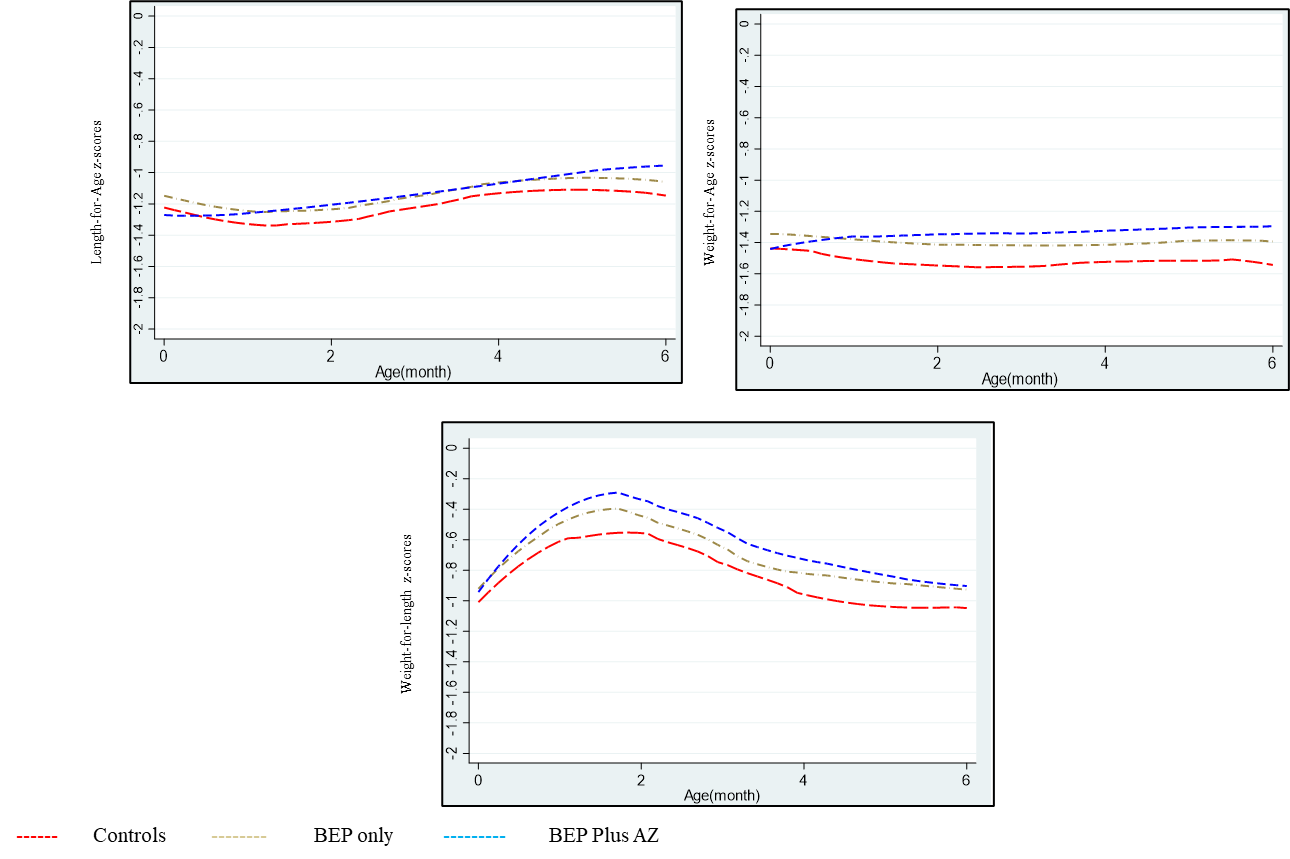


**Supplementary Figure 2 | Trajectories of maternal BMI and MUAC over the period of six months = Controls vs BEP arms***


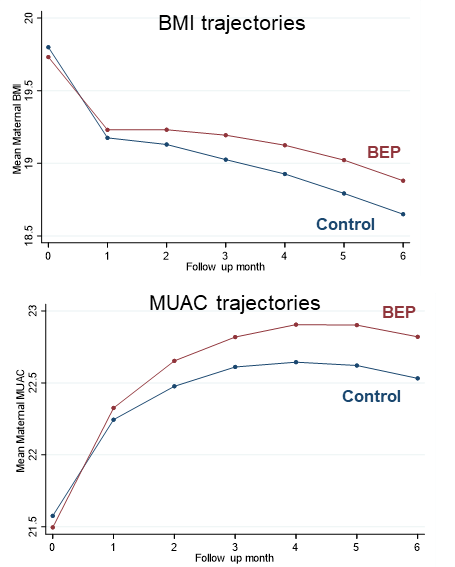


* BEP arm = Combined both intervention arms i.e. BEP only and BEP plus AZ
